# Knowledge, attitude and practice of medical students towards COVID19 in Sudan: A cross sectional study among 19 universities

**DOI:** 10.1016/j.amsu.2022.104874

**Published:** 2022-11-13

**Authors:** Mohammed Alfatih, Khabab Abbasher Hussien Mohamed Ahmed, Radi Tofaha Alhusseini, Elfatih A. Hasabo, Lina Hemmeda, Walaa Elnaiem, Rua Isameldin Bakhiet Mohamed, Monzer Omer Ahmed Abdalla, Khadija ala Abdalmaqsud muhmmed, Osama Mohammed Nowar Taha, Yaman Shurki Adel Husni Yousef, Rawan Raad Hassan Alrufai, Ahmed Emadaldeen Ahmed Mohammed Alamin, Muzamil Musa Mohamed Musa, Saida abdallah mohammed taha abdallah, Mohammed Mahmmoud Fadelallah Eljack, Basaier Mohammed Alamaldeen Kharif, Areej Imad Aldeen Mohamed Idris, Sara Mohamed Abdalla Idris, Mohamed Ahmed Abugibba Mohamed, Malaz Salah Osman Gurashi, Mohammed Alfateh omer Mohammed, Ahmed Bukhari Mohamed Ahmed, Isra Mohamed Hassan Nasr, Abdlrhman saeed mohammed saeed, Mohammed Eltahier Abdalla Omer, Ahmed ElSayed, Mohannad Abdalfdeel Almahie Shaban

**Affiliations:** a Faculty of Medicine, Alzaiem Alazhari University, Khartoum, Sudan; b Faculty of Medicine, University of Khartoum, Khartoum, Sudan; c Faculty of Medicine, University of Gezira, Wad Madani, Sudan; d Faculty of Medicine, Omdurman Islamic University, Omdurman, Sudan; e Faculty of Medicine, Elrazi University, Khartoum, Sudan; f Faculty of Medicine, Sudan University of Science and Technology, Khartoum, Sudan; g Faculty of Medicine, West Kordofan University, Al-Fulah, Sudan; h Faculty of Medicine, Al Neelain University, Khartoum, Sudan; i Faculty of Medicine, Ahfad University for Women, Omdurman, Sudan; j Faculty of Medicine, University of Bakht Alrudam, Ed Dueim, Sudan; k Faculty of Medicine, Dalanj University, Dalang, Sudan; l Faculty of Medicine, Sudan International University, Khartoum, Sudan; m Faculty of Medicine, Ibn Sina University, Khartoum, Sudan; n Faculty of Medicine, University of Medical Sciences and Technology, Khartoum, Sudan; o Faculty of Medicine, University of Science and Technology, Omdurman, Sudan; p Faculty of Medicine, National University, Khartoum, Sudan; q Faculty of Medicine, Shendi University, Shendi, Sudan; r Faculty of Medicine, Karary University, Omdurman, Sudan; s Faculty of Medicine, Nile Vallage University, Khartoum, Sudan; t Faculty of Medicine, Gadarif University, Al Qadarif, Sudan; u Department of Surgery, AlShaab Teaching Hospital, Khartoum, Sudan

**Keywords:** COVID-19, Knowledge, Attitude, Practice, Medical students, Sudan

## Abstract

**Background:**

Since December 2019, an outbreak of severe respiratory infection (COVID-19) emerged in the city of Wuhan in China. The knowledge, awareness and practice of medical students toward COVID-19 pandemic is of most importance as it demonstrates their preparedness to deal with this pandemic. The objective of this study is to assess the knowledge, awareness and practice of medical students in Sudan universities about COVID 19.

**Methodogy:**

This is a cross-sectional study conducted on 19 universities that have medical schools in Sudan. Data from at least 100 medical students from each university were included in the study. Data were collected using an online questionnaire in April 2020. Statistical analysis was conducted using the Statistical Package for Social Science software, version 25.

**Results:**

About 2603 medical students from 19 universities were included. Overall good knowledge and practice were demonstrated by the medical students (88.9%) and (78.6%), respectively. Respondents who answered that the most common clinical symptoms of COVID-19 were the main combination of dry cough, fatigue and fever were (27.7%), and the first initial symptom was headache were (48.3%.). (60.2%) Wear medical masks, (95%) said that avoiding crowded places protects against the spread of COVID-19, and (50.7%) have confidence that Sudan can win the battle against the COVID-19. Finally, (68.8%) agreed that COVID-19 will finally be successfully controlled.

**Conclusion:**

This study has found that medical students in Sudan demonstrated good knowledge and good practice toward Covid19.

## Highlights

• Covid-19 pandemic has captured the attention of the world due to severe political, social, psychological, and economic influences.
• This study has found that medical students in Sudan demonstrated good knowledge and good practice toward COVID-19.

## 1 Introduction

On the January 7, 2020, the Chinese Centre for Disease Control and Prevention (CCDC) isolated the causative agent from throat swab samples, and the name Severe Acute Respiratory Syndrome Coronavirus 2 (SARS-CoV-2) was given to this virus. The World Health Organization (WHO) then renamed it Coronavirus disease-19 (COVID-19) [1]. Coronaviruses are a large family of enveloped RNA viruses that can infect a broad range of animals; including camels, cattle, cats, and bats. In relatively rare events, vectors can transmit coronaviruses to humans, and continued circulation results in human-to-human exposure [2]. COVID-2019 is the third coronavirus emerging in the human population in the past two decades; as preceded by the severe acute respiratory syndrome (SARS) outbreak in 2002 and the Middle East Respiratory Syndrome (MERS) outbreak in 2012 [3,4]. All these coronaviruses have originated in bats [2]. In January 2020, the WHO described the COVID-19 outbreak as an international public health emergency. Thereafter, in March 2020, the WHO announced the disease as a pandemic [5,6].

This pandemic has captured the attention of the world due to severe political, social, psychological, and economic influences, necessitating a strong international concern and collaborative efforts from all countries to prevent the serious spread of COVID-19 [1]. More than 8 million cases were reported in Africa until September 17, 2021, with more than 226 million cases reported worldwide. Since the first occurrence of COVID-19 in Sudan on March 13, 2020, more than 38 000 cases have been confirmed and recorded, with a sum of 2833 lives lost and 31 590 cases recovered, with studies indicating a case fatality rate of 7.7% [2,3]. The firing of case numbers compelled Sudan's government to implement immediate health measures such as isolating infected individuals and implementing personal protective measures. People are advised to avoid overcrowding and maintain proper social distancing. Hand hygiene should also be maintained through frequent hand washing and the use of hand sanitizers. Wearing facemasks and avoiding touching one's face with one's hands have also been shown to effectively prevent the spread of COVID-19 [[7], [8], [9]].

While community perception of these measures remains a source of contention. Only a few studies have been conducted in this area, demonstrating that the potency of government interventional policies was highly reliant on people's adherence to these control measures, which was heavily influenced by their knowledge, attitude, and practice toward COVID-19 [10].

With students returning to their institutions to resume their postponed educational schedules, the seriousness of applying preventive measures can fall back on the overall population as a single case may affect entire students and thus the rest of the community; however, despite this, only a few studies have been conducted in Sudanese medical students to assess their knowledge, attitude, and practice towards Covid-19. We intend to assess medical students' knowledge, attitudes, and practices regarding COVID-19 in 19 Sudanese universities in this cross-sectional study.

## 2 Methodology

### 2.1 Study setting

This cross-sectional study was conducted on universities that have medical schools in Sudan. The included medical schools were chosen according to their willingness to participate. A minimum of 100 medical students from each university were randomly included in this study. Out of 24 medical schools in Sudan, only 19 medical schools met our criteria to be included in this study.

### 2.2 Study participants

Medical students on the selected medical schools constituted our targeted participants. These students were approached by a member of the study team from their own university. The students were asked for their consent to participate, and those who agreed were given a link to the study questionnaire on a Google form. The students were approached on the social media platforms that are widely used amongst students in Sudan (Facebook and WhatsApp). This approach was complemented by telephone calls to stress the importance of our study in some instances. Additionally, the messages on the social media platforms were repeated if the response rate slowed down. Respondents were required to sign in to fill the Google form using their Google account to ensure only one response per participant. During the period from the 7th to the April 18, 2020, all the required responses were collected.

This a cross-sectional study in is fully compliant with the STROCSS 2021 criteria [11].

### 2.3 Data collection method

The study instrument was an online well-structured non-shuffled questionnaire developed by the authors. The questionnaire was initially self-tested by the authors before implementation and a pilot study was done on 40 participants. Our questionnaire was delivered through Google forms questionnaire timed for 3 min and consisted of two parts:

Part one: Demographic data, which had six variables (age, gender, academic year, university name, marital status, and nationality).

Part two: Knowledge, attitudes, and practice, which had 35 questions. Following the guidelines for clinical and community management of COVID-19 by the National Health Commission of the People's Republic of China (24), the COVID-19 knowledge questionnaire was developed by the authors. Further questions about the attitudes and practices towards COVID-19 were added to this questionnaire. This part had 23 questions regarding knowledge of COVID-19. A correct answer was assigned 1 point and an incorrect/unknown answer was assigned 0 points. Making the knowledge score ranges from zero to 23 points, with a higher score denoting a better knowledge of COVID-19. Attitudes towards COVID-19 were measured by two questions and respondents' practice was assessed by 10 questions.

### 2.4 Statistical analysis

Statistical analysis was performed using the using R software version 4.0.2. Data were presented as number (percentages). Chi-square test and fisher exact test were used to find the difference in knowledge attitude and practice of COVID-19 between males and females. A p-value of less than 0.05 was used to determine the level of significance.

## 3 Results

### 3.1 Sociodemographic characteristics

About 2603 medical students from 19 universities responded to the survey (Table 1). As expected, 90.9% were between 18 and 24 years of age with 90.4% of them being between the first and fifth year of medical school, and almost equally distributed through the first to fifth academic years, while those in the sixth year being less represented; this later fact was because 12 out of the 19 medical schools had a 5-year program only. There is only equal representation for the 19 universities. More details could be found on Table 2.

**Table 1:** Distribution of respondents by university.

University	Frequency
Ahfad University for Women	110
AlNeelain University	331
Alzaiem Alazhari University	104
Dalanj University	105
El Razi university	113
Ibn Sina University	106
Karary University	177
Khartoum University	206
National University	103
Nile valley university	126
Omdurman Islamic university	213
Shendi University	105
Sudan international university	114
Sudan University of Science and Technology	129
University of bakht alruda college of medicine	110
University of Gezira	109
University of medical science and technology	101
University of science and technology	103
West Kordufan University	138
Total	2603

**Table 2:** Baseline characteristics of study participants.

Variables	N	Overall, N = 2,740^*1*^	Gender		p-value^b^
			Female, N = 1,741^a^	Male, N = 999^a^	
**Age, years**	2740				**<0.001**
18–20		1046 (38.2%)	739 (42.4%)	307 (30.7%)	
21–22		905 (33.0%)	576 (33.1%)	329 (32.9%)	
23–24		540 (19.7%)	324 (18.6%)	216 (21.6%)	
24–25		165 (6.0%)	75 (4.3%)	90 (9.0%)	
26 or more		84 (3.1%)	27 (1.6%)	57 (5.7%)	
**University**	2740				**<0.001**
Ahfad University for Women		111 (4.1%)	111 (6.4%)	0 (0.0%)	
AlNeelain University Faculty of Medicine		331 (12.1%)	209 (12.0%)	122 (12.2%)	
Alzaiem Alazhari University		104 (3.8%)	49 (2.8%)	55 (5.5%)	
Bahri University		45 (1.6%)	31 (1.8%)	14 (1.4%)	
Dalanj University		105 (3.8%)	71 (4.1%)	34 (3.4%)	
El Razi university		113 (4.1%)	60 (3.4%)	53 (5.3%)	
Ibn Sina University		106 (3.9%)	56 (3.2%)	50 (5.0%)	
Igra college		15 (0.5%)	6 (0.3%)	9 (0.9%)	
International university of Africa		18 (0.7%)	4 (0.2%)	14 (1.4%)	
Karary University		177 (6.5%)	134 (7.7%)	43 (4.3%)	
Khartoum University		206 (7.5%)	135 (7.8%)	71 (7.1%)	
National Ribat University		40 (1.5%)	25 (1.4%)	15 (1.5%)	
National University – Medicine		103 (3.8%)	60 (3.4%)	43 (4.3%)	
Nile valley university		126 (4.6%)	81 (4.7%)	45 (4.5%)	
Omdurman Islamic university		213 (7.8%)	142 (8.2%)	71 (7.1%)	
Shendi University		106 (3.9%)	70 (4.0%)	36 (3.6%)	
Sudan international university		115 (4.2%)	80 (4.6%)	35 (3.5%)	
Sudan University of Science and Technology		103 (3.8%)	73 (4.2%)	30 (3.0%)	
UMST		27 (1.0%)	16 (0.9%)	11 (1.1%)	
University of bakht alruda college of medicine		110 (4.0%)	56 (3.2%)	54 (5.4%)	
University of Gezira		109 (4.0%)	68 (3.9%)	41 (4.1%)	
University of Kassala		16 (0.6%)	6 (0.3%)	10 (1.0%)	
University of medical science and technology		95 (3.5%)	66 (3.8%)	29 (2.9%)	
University of science and technology		108 (3.9%)	67 (3.8%)	41 (4.1%)	
West Kordufan University		138 (5.0%)	65 (3.7%)	73 (7.3%)	
**Level**	2740				**0.01**
1		415 (15.1%)	257 (14.8%)	158 (15.8%)	
2		525 (19.2%)	365 (21.0%)	160 (16.0%)	
3		513 (18.7%)	338 (19.4%)	175 (17.5%)	
4		543 (19.8%)	327 (18.8%)	216 (21.6%)	
5		482 (17.6%)	289 (16.6%)	193 (19.3%)	
6		262 (9.6%)	165 (9.5%)	97 (9.7%)	
**Marital states**	2740				**0.013**
Divorced		4 (0.1%)	3 (0.2%)	1 (0.1%)	
Engaged		132 (4.8%)	95 (5.5%)	37 (3.7%)	
Married		55 (2.0%)	43 (2.5%)	12 (1.2%)	
Single		2549 (93.0%)	1600 (91.9%)	949 (95.0%)	
**From where you get your information about COVID 19? (can choose many answer)**	2740				
social media		2048 (74.7%)	1342 (77.1%)	706 (70.7%)	**<0.001**
News and TV		1663 (60.7%)	1083 (62.2%)	580 (58.1%)	**0.032**
MOH		308 (11.2%)	161 (9.2%)	147 (14.7%)	**<0.001**
WHO update		1682 (61.4%)	994 (57.1%)	688 (68.9%)	**<0.001**
medical journal		803 (29.3%)	443 (25.4%)	360 (36.0%)	**<0.001**
College		444 (16.2%)	234 (13.4%)	210 (21.0%)	**<0.001**

a n (%).

b Pearson's Chi-squared test; Fisher's exact test.

### 3.2 Knowledge

Of all the respondents 74.7% take their covid-19 information from social media, and 29.3% from medical journals. Almost 86.9% Participants responded that the most common clinical symptoms of COVID 19 is dry cough, and 87.2% said it's fever, and about 27.7% indicated dry cough, fatigue and fever as the main combination of clinical symptoms for COVID-19. Also, Respiratory droplets had been identified as the main route of spread of COVID-19 by 94.1% and it was highly associated with the academic year of the medical students with a P value of (< .6), and 86.3% identified that asymptomatic persons cannot spread the virus. 97.2% said that people who contacted someone infected with the COVID-19 virus should be immediately isolated for an observation period of 14 days. (50.9%) have confidence that Sudan can win the battle against the COVID-19. More details about medical students' knowledge could be obtained from Table 3.

**Table 3:** Knowledge and awareness of medical students about COVID-19.

Variables	N	Overall, N = 2,740^*1*^	Gender		p-value^b^
			Female, N = 1,741^a^	Male, N = 999^a^	
**The main clinical symptoms of COVID-19 are? (can choose many answer)**	2740				
Dry cough		2382 (86.9%)	1495 (85.9%)	887 (88.8%)	**0.029**
Fever		2390 (87.2%)	1514 (87.0%)	876 (87.7%)	0.6
Fatigue		1303 (47.6%)	797 (45.8%)	506 (50.7%)	**0.014**
Myalgia		628 (22.9%)	385 (22.1%)	243 (24.3%)	0.2
I don't know		45 (1.6%)	33 (1.9%)	12 (1.2%)	0.2
**What symptoms are uncommon in COVID-19 patients? (can choose many answer)**	2740				
Runny nose		1310 (47.8%)	841 (48.3%)	469 (46.9%)	0.5
Sputum		1405 (51.3%)	893 (51.3%)	512 (51.3%)	>0.9
Sneezing		802 (29.3%)	515 (29.6%)	287 (28.7%)	0.6
Stuffy nose		732 (26.7%)	443 (25.4%)	289 (28.9%)	**0.047**
I don't know		347 (12.7%)	229 (13.2%)	118 (11.8%)	0.3
**First initial symptom of COVID19?**	2740				0.3
Headache		1323 (48.3%)	865 (49.7%)	458 (45.8%)	
I don't know		520 (19.0%)	325 (18.7%)	195 (19.5%)	
loss of smell sensation		277 (10.1%)	180 (10.3%)	97 (9.7%)	
Runny nose		175 (6.4%)	104 (6.0%)	71 (7.1%)	
Sneezing		374 (13.6%)	225 (12.9%)	149 (14.9%)	
Stuffy nose		71 (2.6%)	42 (2.4%)	29 (2.9%)	
**Which COVID-2019 cases are most likely to develop to severe cases? (can choose many answer)**	2740				
Elderly		2290 (83.6%)	1454 (83.5%)	836 (83.7%)	>0.9
Young		169 (6.2%)	112 (6.4%)	57 (5.7%)	0.4
Chronic disease patients		2316 (84.5%)	1475 (84.7%)	841 (84.2%)	0.7
Females		48 (1.8%)	21 (1.2%)	27 (2.7%)	**0.004**
Obese		281 (10.3%)	161 (9.2%)	120 (12.0%)	**0.022**
I don't know		50 (1.8%)	32 (1.8%)	18 (1.8%)	>0.9
**Persons with COVID-2019 cannot infect the virus to others when they don't have a fever**	2740				0.9
FALSE		2364 (86.3%)	1499 (86.1%)	865 (86.6%)	
I don't know		273 (10.0%)	177 (10.2%)	96 (9.6%)	
TRUE		103 (3.8%)	65 (3.7%)	38 (3.8%)	
**Eating or contacting wild animals would result in the infection by the COVID-19 virus?**	2740				0.5
FALSE		793 (28.9%)	495 (28.4%)	298 (29.8%)	
I don't know		564 (20.6%)	370 (21.3%)	194 (19.4%)	
TRUE		1383 (50.5%)	876 (50.3%)	507 (50.8%)	
**When doing normal hospital work wearing general medical masks to prevents infection by the COVID-19 virus?**	2740				0.2
FALSE		611 (22.3%)	375 (21.5%)	236 (23.6%)	
I don't know		147 (5.4%)	87 (5.0%)	60 (6.0%)	
TRUE		1982 (72.3%)	1279 (73.5%)	703 (70.4%)	
**COVID-19 virus spreads via respiratory droplets of infected individuals?**	2740				0.2
FALSE		69 (2.5%)	50 (2.9%)	19 (1.9%)	
I don't know		94 (3.4%)	55 (3.2%)	39 (3.9%)	
TRUE		2577 (94.1%)	1636 (94.0%)	941 (94.2%)	
**Wear general medical masks to prevent the infection by the COVID-19 virus**	2739				0.6
FALSE		8 (0.3%)	5 (0.3%)	3 (0.3%)	
I don't know		1083 (39.5%)	676 (38.8%)	407 (40.8%)	
TRUE		1648 (60.2%)	1060 (60.9%)	588 (58.9%)	
**It is not necessary for children and young adults to take measures to prevent the infection by the COVID-19 virus?**	2740				0.057
FALSE		2346 (85.6%)	1511 (86.8%)	835 (83.6%)	
I don't know		104 (3.8%)	58 (3.3%)	46 (4.6%)	
TRUE		290 (10.6%)	172 (9.9%)	118 (11.8%)	
**To prevent the infection by COVID-19, individuals should avoid going to crowded places such as bus stations and avoid taking busy public transportation?**	2740				0.15
FALSE		75 (2.7%)	40 (2.3%)	35 (3.5%)	
I don't know		63 (2.3%)	38 (2.2%)	25 (2.5%)	
TRUE		2602 (95.0%)	1663 (95.5%)	939 (94.0%)	
**Isolation of people who are infected with the COVID-19 virus is an effective way to reduce the spread of the virus?**	2740				0.2
FALSE		62 (2.3%)	34 (2.0%)	28 (2.8%)	
I don't know		86 (3.1%)	50 (2.9%)	36 (3.6%)	
TRUE		2592 (94.6%)	1657 (95.2%)	935 (93.6%)	
**People who have contact with someone infected with the COVID-19 virus should be immediately isolated in a proper place. In general, the observation period is 14 days?**	2740				0.7
FALSE		29 (1.1%)	18 (1.0%)	11 (1.1%)	
I don't know		47 (1.7%)	27 (1.6%)	20 (2.0%)	
TRUE		2664 (97.2%)	1696 (97.4%)	968 (96.9%)	
**How is the new greeting for the corona-virus?**	2740				0.2
By elbow		2191 (80.0%)	1382 (79.4%)	809 (81.0%)	
By hand		247 (9.0%)	153 (8.8%)	94 (9.4%)	
I don't know		302 (11.0%)	206 (11.8%)	96 (9.6%)	
**Do you think that Corona-virus can spread in hot tropical climate?**	2740				0.078
No		454 (16.6%)	305 (17.5%)	149 (14.9%)	
Yes		2286 (83.4%)	1436 (82.5%)	850 (85.1%)	
**What is most sensitive test for Corona-virus?**	2740				**0.003**
Chest CT scan		613 (22.4%)	397 (22.8%)	216 (21.6%)	
Chest MRI		220 (8.0%)	145 (8.3%)	75 (7.5%)	
Chest X Ray		254 (9.3%)	185 (10.6%)	69 (6.9%)	
RT-PCR (True)		1653 (60.3%)	1014 (58.2%)	639 (64.0%)	
**What is most accurate sample to test for Corona-virus?**	2740				**0.005**
Broncho-alveolar lavage		739 (27.0%)	432 (24.8%)	307 (30.7%)	
CBC		256 (9.3%)	159 (9.1%)	97 (9.7%)	
Nasal swap (True)		1485 (54.2%)	977 (56.1%)	508 (50.9%)	
Sputum sample		260 (9.5%)	173 (9.9%)	87 (8.7%)	
**Do you think that Corona-virus (COVID) 19 can spread by Air? (Yes)**	2740	1482 (54.1%)	952 (54.7%)	530 (53.1%)	0.4
**Do you think that smoking can have a protective effect against Corona-virus (COVID) 19? (Yes)**	2740	311 (11.4%)	207 (11.9%)	104 (10.4%)	0.2
**What is the mortality rate of COVID19?**	2740				**<0.001**
1–2%		309 (11.3%)	190 (10.9%)	119 (11.9%)	
10–20%		458 (16.7%)	349 (20.0%)	109 (10.9%)	
2–3%		808 (29.5%)	488 (28.0%)	320 (32.0%)	
3–5%		721 (26.3%)	417 (24.0%)	304 (30.4%)	
5–10%		444 (16.2%)	297 (17.1%)	147 (14.7%)	
**What is transmission route of Corona-virus (COVID 19)?**	2740				**0.03**
though the respiratory droplets (True)		2300 (83.9%)	1449 (83.2%)	851 (85.2%)	
through contact by skin		273 (10.0%)	183 (10.5%)	90 (9.0%)	
through the body fluids		134 (4.9%)	94 (5.4%)	40 (4.0%)	
through the infected wounds		33 (1.2%)	15 (0.9%)	18 (1.8%)	
**Do you agree that COVID-19 will finally be successfully controlled?**	2740				0.6
Agree		1885 (68.8%)	1210 (69.5%)	675 (67.6%)	
Disagree		389 (14.2%)	241 (13.8%)	148 (14.8%)	
I don't know		466 (17.0%)	290 (16.7%)	176 (17.6%)	

a n (%).

b Pearson's Chi-squared test; Fisher's exact test.

### 3.3 Practice

A total of 72.2% do not go to crowded places most of them were females 78.2%. Females were also more likely to wear masks when leaving home (58.6%) with a P value = <.0001 (significant). Regarding the use of hand sanitizer, (38.1%) said that they always use hand sanitizer after touching foreign surfaces outside their house and 9.3% answered never. Near 93.1% said that they wash their hands with soap first thing when they get back home with females (95.2%) being slightly better. Only 6.5% never used tissue after sneezing, with females being significantly better at using tissues (P value < .001). About 43,1% of females do not shake hands during this COVID-19 pandemic and when asked about frequency of hand shaking it was less among females with most of them answering sometime 70.31% which was significantly different from males with a p-value < 001). In addition, only 27.5% of medical students said that they still hug people. Refer to (Table 4) to explore more.

**Table 4:** Responses of medical students on questions on their practice in COVID-19 pandemic.

Variables	N	Overall, N = 2,740^a^	Gender		p-value^b^
			Female, N = 1,741^a^	Male, N = 999^a^	
**Do you have confidence that Sudan can win the battle against the COVID-19? (Yes)**	2740	1389 (50.7%)	903 (51.9%)	486 (48.6%)	0.1
**In recent days, have you gone to any crowded place? (Yes)**	2740	762 (27.8%)	380 (21.8%)	382 (38.2%)	**<0.001**
**In recent days, have you worn a mask when leaving home? (Yes)**	2740	1526 (55.7%)	1020 (58.6%)	506 (50.7%)	**<0.001**
**Do you use hand sanitizer?**	2740				**0.003**
Always		1043 (38.1%)	693 (39.8%)	350 (35.0%)	
Never		262 (9.6%)	144 (8.3%)	118 (11.8%)	
Sometime		708 (25.8%)	458 (26.3%)	250 (25.0%)	
Usually		727 (26.5%)	446 (25.6%)	281 (28.1%)	
**Do you use hand sanitizer after touching foreign surfaces outside your house? (Yes)**	2740	2122 (77.4%)	1415 (81.3%)	707 (70.8%)	**<0.001**
**Do you wash your hands with soap first thing when you get back home? (Yes)**	2740	2552 (93.1%)	1657 (95.2%)	895 (89.6%)	**<0.001**
**Do you use tissue paper when you sneeze or cough?**	2740				**<0.001**
Always		1164 (42.5%)	832 (47.8%)	332 (33.2%)	
Never		181 (6.6%)	76 (4.4%)	105 (10.5%)	
Sometime		583 (21.3%)	315 (18.1%)	268 (26.8%)	
Usually		812 (29.6%)	518 (29.8%)	294 (29.4%)	
**Do you still shake hands with people after Corona-virus pandemic? (Yes)**	2740	1627 (59.4%)	991 (56.9%)	636 (63.7%)	**<0.001**
**Do you still shake hands with people after Corona-virus pandemic? If yes how frequently?**	2172				**<0.001**
All the time		196 (9.0%)	104 (7.8%)	92 (11.0%)	
most of the time		515 (23.7%)	291 (21.8%)	224 (26.7%)	
sometime		1461 (67.3%)	937 (70.3%)	524 (62.4%)	
**Do you still hug people after spread of Corona-virus? (Yes)**	2740	754 (27.5%)	498 (28.6%)	256 (25.6%)	0.093
**Do you take off your shoes once you get inside the house? (Yes)**	2740	1884 (68.8%)	1210 (69.5%)	674 (67.5%)	0.3

a n (%).

b Pearson's Chi-squared test; Fisher's exact test.

### 3.4 Overall results

We used a rating score to demonstrate the knowledge and practice which was from (100–90) excellent, (89–80) very good, (79–70) good, (69–60) faire, (59–50) borderline and below (50%) fail. This study has found that medical students in Sudan demonstrated very good knowledge (88.9%) and good practice (78.6%) toward Covid-19.

## 4 Discussion

With the emergence of COVID-19 from the city of Wuhan, China in 2019 [1,2] and its rapid spread around the globe with more than 23 000 cases in Sudan, more than 2.5 million cases in Africa and more than 80 million cases around the globe until the end of December 2020 [9,12], knowledge, awareness and practice (KAP) about COVID-19 among medical students is highly crucial. This study set out with the aim of assessing the knowledge and awareness and knowledge of medical students in Sudan concerning the novel corona virus COVID-19.

In this cross-sectional study, we provided an insight of knowledge and practice towards COVID-19 among medical students during the first wave of the pandemic. This study showed that the knowledge score among medical students in Sudan, was an overall good knowledge of (88.9%), in addition about (78.6%) of the participants had good practice. These findings are similar with that of Sonam Maheshwari (2019) in their study among medical students in the government medical college in Uttarakhan, India, which demonstrated good knowledge (92.7%) and about (80%) in the practice of the respondents [13], We attribute this good level to the source of information that medical students get their information from, with social media being the most common source (74.7%), news and TV (60.7%), WHO updates (61.4%), medical journals (29.3%) and ministry of health updates (11%.2). These sources are somehow similar to the results of phan LT et al. which found that social media was the commonest source (34%) [14].

Overall, there was no significant difference in Knowledge score between gender males and females. In the practice aspect females demonstrated better practice in using hands sanitizers, masks and not going to crowed places. Finally, all medical students think that Sudan can win the battle against COVID-19.

The limitations of the study hopefully have no or minimal impact on the study results. As its a cross-sectional study it doesn't assess cause, change overtime, and relationship. Moreover, data collection was in the form of self-administered questionnaires so there is a risk of information bias. On the other hand, the study included 19 universities so reflecting the overall knowledge of Sudanese medical students with generalizable findings and up-to date information that improve preventive measures for COVID-19.

## 5 Conclusion

This study has found that medical students in Sudan demonstrated good knowledge and good practice toward COVID-19. Although the results were very positive, further education and awareness should be carried out to increase the preparedness of medical students toward such pandemics and public health modules should focus more on the importance of the knowledge of newly emerging diseases and the practices towards them.

## Ethical approval

Ethical clearance was obtained from the National Health Research Ethics Committee, Federal Ministry of Health of Sudan (Ethical number: 4-5-20) before conducting data collection.

## Ethical declaration

Ethical clearance was obtained from the National Health Research Ethics Committee, Federal Ministry of Health of Sudan (Ethical number: 4-5-20) before conducting data collection. At the time of data collection written consent was obtained from the study participants, each participant was asked to check a question asking about his free will to participate in the study, the obtained information was confidentially handled and processed through all the research period. All the costs of doing this study were borne by the authors who had no conflict of interest.

## Funding

The authors received no specific funding for this work.

## Author contribution

Study design, data collection, results interpretation, and writing: Mohammed Alfatih, Khabab Abbasher Hussien Mohamed Ahmed, Elfatih A. Hasabo, Lina Hemmeda, Walaa Elnaiem.

Study design, data analysis, ethical clearance acquisition, and writing:, Radi Tofaha Alhusseini, Rua Isameldin Bakhiet Mohamed, Monzer Omer Ahmed Abdalla, Khadija ala Abdalmaqsud muhmmed, Osama Mohammed Nowar Taha, Yaman Shurki Adel Husni Yousef, Rawan Raad Hassan Alrufai, AHMED EMADALDEEN AHMED MOHAMMED ALAMIN, Muzamil Musa Mohamed Musa, Saida abdallah mohammed taha Abdallah, MOHAMMED MAHMMOUD FADEL ALLAH MOHAMMED, Basaier Mohammed Alamaldeen Kharif, Areej Imad Aldeen Mohamed Idris, Sara Mohamed Abdalla Idris ^13^, Mohamed Ahmed Abugibba Mohamed, Malaz Salah Osman Gurashi, Mohammed Alfateh omer Mohammed, Ahmed Bukhari Mohamed Ahmed.

Study design, data collection, revising and editing the drafted manuscript: Isra Mohamed Hassan Nasr, Abdlrhman saeed mohammed saeed, Mohammed Eltahier Abda lla Omer, Ahmed ElSayed, Mohannad Abdalfdeel Almahie Shaban.

## Registration of research studies

Name of the registry: Not applicable.
Unique Identifying number or registration ID: Not applicable.
Hyperlink to your specific registration (must be publicly accessible and will be checked): Not applicable.

## Guarantor

Lina Hemmeda.

## Consent

At the time of data collection written consent was obtained from the study participants, each participant was asked to check a question asking about his free will to participate in the study, the obtained information was confidentially handled and processed through all the research period.

## Provenance and peer review

Not commissioned, externally peer reviewed.

## Declaration of competing interest

The authors declare that there are no competing interests.
